# How social workers are positioned and constructed as contributors within national palliative care policies in Sweden: a policy analysis

**DOI:** 10.1177/26323524241289601

**Published:** 2024-10-17

**Authors:** Axel Ågren, Bodil Holmberg

**Affiliations:** Division of Social Work, Department of Culture and Society (IKOS), Linköping University, Campus Norrköping, Linköping SE-601 74, Sweden; Department of Health and Caring Sciences, Linnaeus University, Växjö, Sweden

**Keywords:** palliative care, policy analysis, professionalization, role blurring, social work

## Abstract

**Background:**

Palliative care is expected to acknowledge physical, psychological, emotional, spiritual, and social needs, to which social workers can contribute with expertise on recognizing the importance of social relations and how social inequalities impact on individuals. The social work profession faces challenges in claiming their contributions in the hierarchies of medical professions. Polices play an important role in constructing and positioning responsibilities of professions.

**Objectives:**

To analyze how social workers are positioned as contributors to palliative care within national-level policies in Sweden.

**Methods and materials:**

A policy analysis was conducted through a constructivist lens. Data were collected from 10 Swedish national-level policy documents on palliative care.

**Results:**

Three themes emerged: “A natural presence conveying special expertise”; “Emotional conversational support before and after death”; and “Practical support.” Overall, social workers’ contribution was vaguely described and in similar ways as registered nurses.

**Conclusion:**

In Swedish policies analyzed, social workers’ contributions were difficult to differentiate from that of registered nurses, which may complicate their mutual collaborations and cause confusion for individuals at the end of life and their next of kin. Future research on how different professions are positioned within palliative care is needed to reduce risks of role blurring between professions with similar aims. Moreover, given social workers long history of psychosocial knowledge, meanings of this concept need to be further acknowledged.

## Introduction

The overarching policy goal in Sweden is that palliative care should be universal, carried out in all care contexts, for all diseases and medical conditions, and that the care should be equally distributed throughout the country.^1^ Moreover, palliative care in Sweden is mostly built upon the founding ideas of the hospice care philosophy, acknowledging the physical, psychological, social, emotional, and spiritual needs of the dying.^2,3^ Cicely Saunders, founder of the hospice movement, embodied this holistic approach, being a physician, nurse, and social worker by training, combined with a religious faith and with a strong determination to improve knowledge on pain relief for dying patients.^4^ These ideologies stemmed from a critique of how the institutionalization and professionalization of death and dying led to the wishes, needs, and social aspects of dying patients becoming neglected in hospital settings, due to the focus on medical aspects of the dying.^5^ Thus, this critique served as a point of departure for the hospice movement and the subsequent development of palliative care.^6,7^

Although critically scrutinized by scholars,^8,9^ the notion of Western countries as being death denying has been present for decades in both public^10,11^ and professional discourse,^12^ where it is repeatedly argued that more knowledge about death and dying is needed.^13,14^ The need for more knowledge and expertise on the social aspects of dying which social workers can contribute to is repeatedly emphasized.^15^ However, studies have illustrated how palliative care expertise is negotiated and located within the world of medicine, where rationality, technical expertise on symptom relief, evidence-based treatments, and universal concepts are means for gaining legitimacy and resources.^16,17^ Thus, the social work profession has been found to face challenges when claiming its knowledge contribution in the hierarchy of healthcare and medicine.^18^

How palliative care is organized regarding problems, solutions, and responsibilities is to a great extent dependent upon how the issue is constructed in national-level policies.^19,20^ Thus, policies play an important role in the allocation of resources, knowledge provision, support to professions, the development of methods, and how different professions are positioned as contributors within a specific context. Meanwhile, a necessary component of policies is to address policy problems in digestible ways that gain legitimacy, both in politics and among the public.^21^

Sweden serves as an example of a country where policies play a key role in the organization of palliative care,^19^ and there is a need for more research on how social work is positioned within this context. Accordingly, the aim of this study is to analyze how social workers are positioned as contributors to palliative care within national policies in Sweden. In line with this aim, we have analyzed the empirical material based on the following research questions:

- What tasks are social workers expected to perform within palliative care settings according to national policies?
- How are social workers in palliative care settings supposed to perform their assignments according to national policies?

### Social work within the healthcare sector in Sweden: A brief overview

Social work has been part of the Swedish healthcare sector for over 100 years, providing staff in the role of counselors, who originally focused on the social and economic situation of the “mentally deranged,” defending their rights, preparing them for a life outside institutions, providing body care, supporting patients and relatives, and cooperating with private and public charities.^22^ Initially, both nurses and those with a social education were able to practice this profession. When the Swedish Counselors Association was founded in 1944, it was decided that a degree from the Social Institutes (at the time a post-secondary occupational education) would be the requirement. With the breakthrough in the fields of mental hygiene and the psychological and psychoanalysis movements, dialogue with patients and relatives became a method employed by the profession. The individualistic orientation led to intense debates and an identity crisis within the profession during the 1960s and 1970s, with a tension emerging between individualistic and structural understandings of social problems, which is still present today. Today, the counselor profession is certified and can be achieved through academic studies on master level after a bachelor’s degree in social work,^23^ with focus on psychosocial perspectives to understand an individual’s relationships and social environment^22,24^ and are involved in various healthcare contexts, such as palliative care.

### Contemporary Swedish palliative care

The first palliative care policies published in Sweden, in the 1970s, were dominated by psychological perspectives, criticizing the lack of competences to address the psychological and existential issues in end-of-life care, leaving individuals to die with unanswered questions in “total loneliness” and in unfamiliar institutional settings.^25^ Hence, it was argued that a human connection was important, provided through trained staff. The task was to increase knowledge on the psychology of dying and educate staff (whose profession was often not specified) in how to provide psychological end-of-life care.^19^

Although the intention is to provide care for all age groups, palliative care is continuously prioritized for younger people with terminal cancer,^26^ who easily gain access to care from specialized, multiprofessional teams, of which social workers are a natural part.^27^ However, populations are ageing in Sweden as all over the world, and worldwide, the number of persons aged 80 or above is assumed to triple between 2020 and 2050.^28^ Thus, death and dying are increasingly becoming issues of later life, and it is common for older adults with multiple and chronical diseases to die on hospital wards specializing in oncology or geriatrics, with access primarily to general palliative care, meaning that staff are supposed to have a basic knowledge of palliative care. Further, older adults increasingly die in their homes or in nursing homes, where it is desired but not always a fact that staff can employ a palliative care approach, conveying the integration of palliative care methods and procedures. General palliative care and end-of-life care with a palliative approach are usually provided by hospital nurses, physicians, and assistant nurses, as well as by community nurses, nursing-home nurses, with typically no social workers being employed.^29^ This type of care is assumed to grow in Sweden, due to a recently launched governmental inquiry called *God och nära vård* (the English term for the governmental inquiry is *Good quality, local health care*), which aims to reform healthcare by transferring more of the care from hospitals to primary care settings, that is, the patients’ homes or nursing homes. The goal of this care reform is to provide a more person-centered care by allowing patients to stay in well-known surroundings but requires an increased degree of collaboration between various care instances, and a continued development of specialized and mobile palliative home-care teams.^30^ In Sweden, specialized palliative teams can be consulted by basic care settings, but a recent report from the Swedish Cancer Foundation^31^ reveals that access to such teams is unevenly distributed due to geographical variations across the country, regarding both common palliative care and specialized palliative care. The report also reveals inequalities by diagnosis, with patients diagnosed with cancer generally having better access to palliative care compared to patients with other diagnoses. They also found that there is a lack of political governance and coherent infrastructure for palliative care, a shortage of staff with adequate palliative competence, and a lack of organized continuous training for them.^31^ In alignment with this, a study reveals that Swedish politicians, chief medical officers as well as other hospital staff possess insufficient knowledge of palliative care policies and perceive the hasty hospital culture as an obstacle to implementing them.^32^ Thus, older patients suffering from diseases other than cancer may face death without access to the qualified psycho-social, emotional, and practical support that can be provided by a social worker.

## Methods and materials

### Design

We draw on Berger and Luckmann’s argument that social constructionism should shed light on how various issues in society become taken for granted as “knowledge.”^33^ Inspired by a social constructionist perspective on policy analysis, we concur with Bacchi^34^ in that governments play an active role in constructing policy “problems” in specific ways through public policies. This results in shaping certain understandings of the “problem,” which then legitimize specific types of responses to the “problem” at hand.^34^ In this study, the policy problem in focus is how social workers are positioned as contributors to palliative care.

### Data collection

The empirical material gathered for this study consists of national policy documents on palliative care in Sweden, published during the period 1977–2023. The motive for this demarcation is that the first policy document on palliative care (at the time defined as terminal care) was published in 1977 and that policy documents are still published today. The relevance of this time period is further motivated by a historical exposé on the development of palliative care in Sweden, illustrating how policies have evolved over this time period.^35^ The Swedish online database Libris, which collects all publications from the collections of Swedish university libraries, was used to find these documents.^36^ The search words were *vård i livets slutskede* (end-of-life care) and *palliativ vård* (palliative care) and were applied to documents published by state agencies, governmental reports, publications with the aim of serving as national policies and two dissertations. These searches led to finding 10 publications. An overview of the empirical material is provided in Table 1.

**Table 1.** Policy documents regulating Swedish palliative care.

Dissertations	Swedish NBHW	Government policies	Care policies
Terminal care, a method for psychological care of dying cancer patients by Feigenberg.^37^	End-of-life care. The NBHW’s assessment of the development in county councils and municipalities.^39^	Death concerns us all: Dignified end-of-life care (SOU,^1^ p. 6).	National Care Program, RCC.^44^
Patients’ reactions to impending death: A clinical study by Qvarnström.^38^	Research reflecting end-of-life care: Compilation of current research.^40^	End-of-life care (SOU,^47^ p. 59).	
	National knowledge support for good palliative care at the end of life: Guidance, recommendations and indicators. Support for governance and management.^43^		
	Palliative care at the end of life: summary with areas for improvement.^41^		
	National guidelines: evaluation 2016 palliative care in the final stage. Indicators and basis for assessments.^42^		

NBHW, National Board of Health and Welfare; RCC, Regional Cancer Centre in Cooperation.

**Figure 1.**
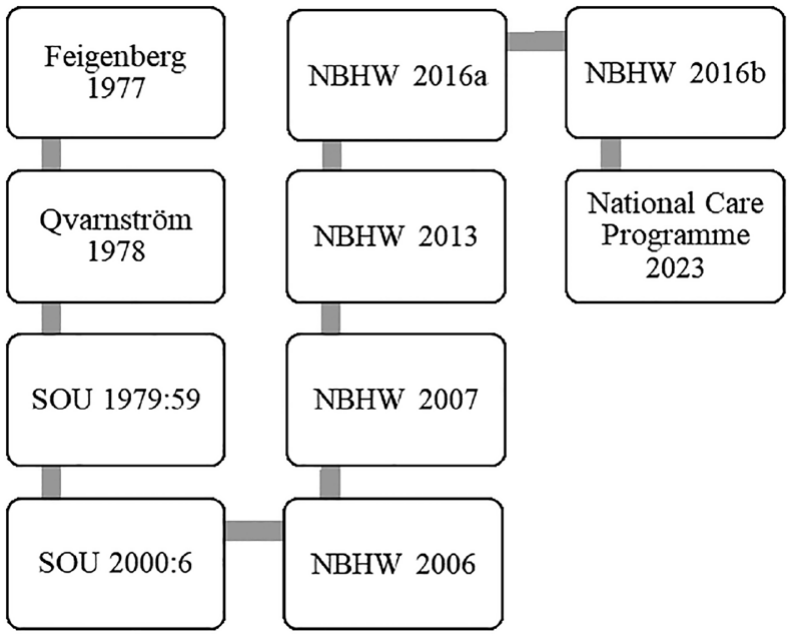
Timeline describing policy document development.

These documents have evolved over the course of several decades, starting with the publication of two dissertations, one by a psychiatrist^37^ and the other by a registered nurse,^38^ to which later documents often refer. One example of how the documents are linked is illustrated in the Swedish governmental report,^1^ which was followed by several documents from the National Board of Health and Welfare, which were given the task of continuously regulating and investigating Swedish palliative care.^39–43^ Recently, care professions within palliative care have been involved in developing policy documents regarding how to provide palliative care in a national care program published by Regional Cancer Centre in Cooperation (RCC)^44^ (see Figure 1).

### Data analysis

Initially, the policy documents, comprising 1325 book pages of text, were read through for the purposes of familiarization and identification of text units corresponding to the study’s aim. Thus, we sought for text units describing *what* social workers are expected to do in palliative care settings, and *how* they are supposed to do it. The identified text units were coded and collected in a separate document to provide an overview. After this process, the textual data, consisting of extracted text units corresponding to the study’s aim, comprised nine (A4) pages of singe-spaced text. Thereafter, the coded text units were repeatedly read through. Subsequently, they were arranged into groups, and further re-arranged into themes and sub-themes representing common elements. The method used was content analysis according to Krippendorff^45^ (see Table 2).

**Table 2.** Themes and sub-themes.

Themes	Sub-themes
A natural presence conveying special expertise	Natural member of the palliative team Holder of special expertise
Emotional conversation support before and after death	Counseling support to patients and next of kin Emotional conversation support for next of kin after death Support for co-workers
Practical support	Arrange practical help for patients and next of kin External consultation

To support the study’s trustworthiness, we attempted to reach high degrees of dependability, credibility, confirmability, and transferability.^46^ Accordingly, dependability and credibility were ensured by an ongoing discussion between the authors, but also by peer debriefing, where independent researchers scrutinized the analysis and manuscript, able to elucidate and decrease the impact of the authors’ pre-understanding. Confirmability was ensured by a careful description of the analysis process, whereas transferability was facilitated by a thorough description of the context, as well as the use of excerpts from policy document in the findings section.

Throughout the process of analysis, we have been guided by social constructionist perspectives on policy analysis, with the objective of dismantling objects that are taken for granted and illustrate how they have gained the status of “truths” that serve to govern societies, individuals, and professions.^34,33^ This methodological stance is supported by Krippendorff,^45^ who claims all contexts to be constructed by someone, thus difficult to objectify. When analyzing the empirical material for this study, our attention has been drawn to how social workers are positioned as contributing to palliative care. In terms of analyzing “problems” in the policy documents, we have searched for formulations regarding the involvement of social workers, their actual working tasks, and their practical contributions to palliative care.

## Results

In this segment, we present our results after having analyzed policy documents on palliative care in Sweden (see Table 2). We found that the issue of multiprofessional teams was highlighted as important throughout the policies over time. In the first governmental report on palliative care (at the time defined as end-of-life care), the need for multiprofessional teams was presented as follows:

A work organization based on teams provides better conditions, given that the care team and others who participate in the work with the patient must have the same information about the patient, thus gaining an increasing awareness about what information is regarded suitable to pass on to the patient.^47^ (p. 180)

Hence, working in multiprofessional teams is addressed as unequivocally positive and a primary goal when deciding how palliative care should be organized. Little attention is paid, however, to the roles and contributions of the various professions within these teams.

Moving forward to the governmental report from 2001, the importance of involving several professions is underscored as: “*Collaboration in a multi-professional team (i.e. a team in which doctors, nurses, assistant nurses, etc. are included)*” should constitute one of four future cornerstones of palliative care. Furthermore, within the cornerstone *communication and relations*, communication within care teams is emphasized.^1^ In 2023, although not made explicit how multiprofessional teams should be organized and what tasks social workers should perform, this way of organizing palliative care is still presented as the major solution to how palliative should be organized and improved.^44^ This illustrates a recurring finding in our analysis of the policy documents; formulations are often general and *could* include social workers, but often do not explicitly state the contributions social workers should make within palliative care.

### A natural presence conveying special expertise

The Swedish policy documents position social workers as natural members of the palliative care team. However, social workers expertise is not defined in the policies analyzed.

#### Natural member of the palliative team

Regarding social workers as a natural part of the multiprofessional palliative team, the Swedish policy documents advocate for them to deliver the psychosocial support for complex needs that might be required to preserve patients’ and next of kin’s dignity and quality of life. However, it is not made explicit what tasks are supposed to be performed by social workers, as the texts speak of assignments for the palliative care team in general:

A fundamental idea is that combinations of professions and competencies should result in collective knowledge to solve complex problems and achieve greater efficiency, with more satisfied patients and relatives.^44^ (p. 67)

Thus, the presence of social workers is expected as a natural element in the palliative team.

#### Holder of special expertise

The policy documents describe it as important that social workers receive continuing education and guidance, to preserve their specialist palliative competence and enable them to remain an important member of the palliative team:

Several professions are needed to provide dignified care at the end of life, for instance physicians, nurses, paramedics, dieticians, and social workers. Such a breadth of competencies requires a solid theoretical foundation.^39^ (p. 29)

Nevertheless, the policy documents do not specify what this continuing education and theoretical foundation should include.

### Emotional conversational support, before and after death

The Swedish policy documents define social workers as having the competence to support patients and next of kin through conversations. As formulated in the policies, this task can, however, also be achieved by other professionals, such as psychologists.

#### Counseling support to patients and next of kin

Throughout the documents, it is seen as valuable for patients to receive emotional support through existential conversations. According to the policy documents, these conversations should be led by members of the palliative care team, who act as counseling support. To further deal with sadness, anxiety and worry, it is emphasized that patients can summarize and reflect upon their lives with, for example, a social worker, as stressed in this quote:

It is required that relatives receive both verbal and written information about where you can turn to when something urgent or unforeseen occurs, regardless of the time of day. The next of kin should be offered professional help, from a social worker, a psychologist, or a spiritual representative.^39^ (p. 58)

Thus, a social worker is expected to take care of patients and next of kin who present with needs that require special competence. Meanwhile, as stated in the excerpt, this task could just as well be performed by a psychologist or a spiritual representative.

If the patient or next of kin asks for it, or if their reaction patterns deviate significantly from a normal stress response, it is stipulated that they need to be referred to a social worker, a psychologist or a nurse with psychotherapeutic education for support. It is not specified what characterizes such a deviation, or which profession is assumed to differentiate normal from abnormal reactions.

#### Emotional conversational support to next of kin after death

The issue of support for next of kin after death has occurred is also addressed in policies, stating that:

The grief support to next of kin can be provided at various levels, depending upon their need. [. . .] support is given when grief has become complicated. Complicated grief can be treated in a cognitive way, whereby next of kin find ways to deal with emotions and memories.^44^ (p. 51)

Hence, if the next of kin’s grief becomes complicated, they may need help to intervene in their grieving process and/or receive cognitive treatment from specifically trained professionals, such as a social worker, or a psychologist. Again, social workers are mentioned but grouped together with other professions.

#### Support to co-workers

According to the Swedish policy documents, social workers are also expected to support, advise, guide, and educate their palliative team co-workers and to help them handle the various challenges in their work in a professional manner.

### Providers of practical support

In the Swedish policy documents it is stated that social workers may provide different types of practical support, often to be performed in collaboration with professions, such as nurses.

#### Arrange practical help for patients and next of kin

According to the Swedish policy documents, social workers can assist patients and next of kin in their daily life, for instance regarding childcare, to arrange a religious rite, to gain access to suitable and stimulating creative activities, or to get in contact with the authorities:

Qualified psychosocial support is carried out by a social worker and covers several areas, including both emotional and practical support, and the areas often overlap. This may involve practical social measures, information, counseling, and referral, as well as psychosocial conversations to support change.^44^ (p. 42)

What appears here is that social workers are perceived as possessing both practical and emotional expertise which is of relevance for a palliative team. Moreover, in one segment of a policy document, social workers from palliative care teams are positioned as active in areas covered by general palliative care, but also when the care needs are very specific:

It is important to establish contact with a social worker, a nurse with special training, or a psychologist when there is a need to investigate the risk of suicide.^44^ (p. 36)

This entails social workers making home visits that include specific operational interventions, mostly in collaboration with a nurse. These distinct ways of explicitly stating social workers contributions deviates, thus, from the overall finding that the contributions of social workers are constructed vaguely.

## Discussion

The aim of this study was to analyze how social workers are positioned as contributors to palliative care in Sweden within national policies. A key finding of this study was that social workers are described as part of palliative care, mainly through their involvement in multiprofessional teams. According to the policy documents, social workers are expected to aid patients and next of kin in relation to practical arrangements, such as getting in contact with the authorities. The actual contribution of social workers and what tasks they should perform was, however, overall not specified. One example emerges from the RCC^44^ policies, where counselors (who, in Sweden, are educated social workers) were positioned as having a central role in providing psychosocial support. Details regarding how, when, and where this support could or should be provided is, however, not explicitly formulated. The actual content and meaning of psychosocial support, and why social workers should provide this support, is only briefly mentioned. These findings resonate with other policy studies, arguing that policies are often characterized by being ambiguous, vague, and having overly positive expectations.^48^

Within the policies analyzed, multiprofessional teams had the status of a taken-for-granted ideal solution to problems within palliative care. We traced back the underlying notion of this ideal to the recurring notion of dying as complex and in need of understanding from different perspectives, not only from the viewpoint of the physician. Social workers were positioned, both implicitly and explicitly, as part of this team. How these teams were to be organized, and what the various involved professions should contribute with and be responsible for was not stipulated and suggestions for the implementation of these ideals were not addressed. These findings are echoed in Whitelaw et al.’s^48^ review of the term “policy” in the palliative care literature, stating that palliative care policy narratives are aspirational, exhortative, and normative, advocating how palliative care *should* be organized, with less attention on *how* policies might be implemented. Such vague formulations could be explained by the purpose of the policies analyzed, where national policies seemingly have a general tone, leaving it to specific units to adapt policies in accordance with local and organizational conditions.

This vagueness was also found in policies on specialized palliative care teams, which endorsed the suggestion that these should consist of at least one physician and one nurse, and preferably also have psychosocial competence, through the inclusion of a social worker or a psychologist.^44^ Apart from the general vagueness of policies, as also found in other studies, it is difficult to grasp why social workers are addressed in such vague terms. The policy documents do not provide an explanation for this vagueness, but this may have historical roots found in for instance writings by Dame Cicely Saunders. Despite being trained as a physician, nurse, and social worker herself, one part of her publications addressed physicians, the second part highlighted nurses, while the last did not address social workers, but instead the public.^49^ This underlines a paradox that persists today, where social workers and social perspectives are embraced, but simultaneously placed in the periphery in the hierarchy of medical professions through vague formulations.

We found the total number of texts explicitly dealing with social workers’ contributions to palliative care to be limited. There could be several reasons for this. First, policies need to reflect the context in which they are engaged, and palliative care in Sweden is mainly located in the realm of medicine and healthcare, perhaps not sufficiently recognizing the growing context of older patients with multiple and chronic diseases, who die at home or in nursing homes.^31^ Of course, policies could encourage change, and encourage contributions of social work. It is, however, argued that palliative care has been subject to biomedicalization through the emergence of biomedical technoscientific interventions and increases in medical scientific knowledge production^3,50^ as a means of gaining financial resources and distinctively defining what palliative care is within the medical world.^17^ Moreover, specialist training, clinical trials, and statistical analyses within medicine and healthcare have altered how we perceive our bodies, lives, death and dying and, consequently, how palliative care should be conducted.^3,51^ Hence, attempts to define what palliative care is and efforts to gain legitimacy are primarily taking place within healthcare and medicine policies and seemingly not within the context of social work policies.

Issues of death and dying are found to be addressed only to a minor extent within social work educations.^52^ One suggested way of highlighting this issue is to specifically educate social workers during their training, not only in social care, but also in palliative care and teach them to identify and report palliative patients’ and next of kins’ needs, not the least within the field of chronic and life-limiting diseases. The education should also entail practice in exchanging information and develop care plans in close collaboration with other palliative care team members. Added to this, social workers within palliative care contexts should gain skills in notions on death and dying related to religion, cultures, and characteristics of various life spans. They should also learn more about theories on grief and be taught how to practically provide psychoeducation to patients, next of kins and other members of their palliative care team.^53^

A crucial question when conducting policy analysis is how the policy problem at hand is represented.^34^ In the policies analyzed, the foundational problem was that the needs of the dying are not acknowledged due to lack of knowledge, caused by denial of and unwillingness to deal with death and dying, both within the healthcare sector and across society. Hence, policies’ primary point of departure was on highlighting deficiencies, which is also found in other policy studies, which according to Whitelaw et al.^48^ may potentially lead to defensive responses by designated professions. The overarching solution to these problems, as presented in policies, was, as in the case of palliative care policies in the United Kingdom,^54^ through improving compassionate end-of-life care within healthcare. The policy problem and its solutions were, consequently, allocated in the context of healthcare and not social work. In comparison with the contributions of social workers, the policy documents frequently describe the nurse of the palliative team as being obliged to provide compassionate emotional support to patients and to next of kin by being present, interested, and a good listener. Social workers are also seen as providing compassionate care, but this was not elaborated in the same extensive ways as in the case of nurses. These descriptions may be confusing to the professionals reading these policy documents and may raise questions regarding which profession is assumed to provide psychosocial support. Adding to this, the International Council of Nurses^55^ has declared that nurses are expected to work from a holistic perspective, thereby attending to physical, psychological, social, and existential needs, while also ensuring the human rights of patients and their families. This holistic approach also applies to social workers as found in contexts such as the Global Agenda^56^ and educations of social workers.^22^ Adding to this vagueness, a study on American social workers who were experienced in working with seriously ill patients describe their most needed competence as to “use best practices to assess patients and next of kins based upon a biopsychosocialspiritual approach.”^57^ This means that nurses and social workers may perceive their main task in similar ways, potentially leading to “role blurring”^58^ and situations of confusion and tension, when social workers and nurses disagree about who should be responsible for what. This may lead to situations where both professions attend to the patient’s and next of kin’s issues in their own way, unaware of the efforts made by the other profession.

### Strengths and limitations

The authors of this study possess different competencies; the first author is a social scientist within social work, while the second author is a registered nurse with long experience of working within public palliative care contexts. This conveys a possible weakness, as both authors carry experiences that may have impacted the analysis in unconscious ways. However, this interdisciplinary collaboration may also be considered as a strength, enabling analysis from different perspectives, with the finding that nurses’ and social workers’ overall tasks were constructed in very similar ways. The two perspectives have also helped the two researchers to curb, and sometimes question, their own preconceptions, which has contributed to the study’s credibility.^46^ After having read all the relevant policy documents, we found that very few texts addressed social workers. This may potentially be seen as a weakness. On the other hand, the sparsity of data is a finding, highlighting that the contributions of social workers is not much described in Swedish policy documents.

A limitation worth highlighting is that we cannot guarantee that we have covered all relevant policy documents. Within for example eldercare, oncology, and other disease-specific policies, the issue of how end-of-life care should be organized can be assumed to be mentioned. However, since focus in this study is on policies within the realm of palliative care with explicit aims of influencing how this context is organized, we have only included documents with these specific aims. After extensive reviewing of the policy field of palliative care, dialogues with experts within this field, and previously conducted studies, we consider that we cover all relevant documents.

## Conclusions and implications

The way in which Swedish policies are formulated has implications for how palliative care is organized and conducted. Meanwhile, since palliative care involves several professions, it is important to highlight how different professions are positioned as contributing to this context. Overall, in both research and policies, social workers’ contributions of providing psychosocial and practical support, acknowledging the individual’s relationships with the surrounding society, and their attempts to ensure social justice and expertise in cooperating with other professions are much sought after, but policies are vague in describing actual contributions, and working tasks that should be conducted by social workers are often formulated in similar ways as those of registered nurses. This is particularly important in contemporary Sweden, where a reform transferring more care from hospital to patients’ home is recently launched, requesting a further development of specialized and mobile palliative home-care teams. Simultaneously, recent reporting reveals inequalities regarding access to palliative care, where older non-cancer patients living in sparsely populated areas may have reduced access to palliative care team competence. As older populations are growing worldwide, palliative care increasingly becomes an issue of later life. This may convey a global need of clarifying the expected contributions of social workers within this expanding care context. Hence, in order to strengthen the importance of social work within palliative care, it is important to further highlight social workers’ contributions to this care context. Consequently, an issue for future scientific inquiry is to compare occupational descriptions of the professions involved in palliative care, to elucidate similarities and differences in how the involved professions are positioned and the contributions they are expected to make within palliative care. Furthermore, considering the long history of psychosocial knowledge and expertise of social workers, meanings of this concept within palliative care need to be further examined and acknowledged.

## Declarations

**Ethics approval and consent to participate:** No humans participated in this study. Therefore, there was no need of ethics approval.

**Consent for publication:** Not applicable.

**Availability of data and materials:** All material analyzed are public and thus available to all.
